# Validation of the Moroccan Arabic Version of the Pediatric International Knee Documentation Committee Score (Pedi-IKDC) Questionnaire for Children With Knee Disorders

**DOI:** 10.7759/cureus.36391

**Published:** 2023-03-20

**Authors:** Soumaya Benmaamar, Abderahim Kamli, Ibtissam El Harch, Nabil Chettahi, Noura Qarmiche, Nada Otmani, Nabil Tachfouti, Mohamed Berraho, My Abderrahmane Afifi, Samira EL Fakir

**Affiliations:** 1 Laboratory of Epidemiology, Clinical Research and Community Health, Faculty of Medicine and Pharmacy, Sidi Mohamed Ben Abdellah University, Fez, MAR; 2 Pediatric and Orthopedic Surgery Department, Hassan II University Hospital, Fez, MAR

**Keywords:** morocco, arabic, psychometrics tests, pedi-ikdc, validation

## Abstract

Background

The Pedi International Knee Documentation Committee (Pedi-IKDC) is a questionnaire for the evaluation of knee function in children and adolescents with knee disorders. It has been translated and validated into many languages. The aim of this study was to translate this questionnaire into Moroccan Arabic and evaluate its psychometric properties in a pediatric population.

Methods

The original English version of the questionnaire was translated into Moroccan Arabic according to international guidelines. The Arabic version was administered twice to two groups: a group of children with knee disorders and a control group, and the following properties were calculated: reliability, internal consistency, and discriminant validity. The reliability was assessed using the intraclass correlation coefficient (ICC), standard error of measurement (SEM), and smallest detectable change. Internal consistency was evaluated using Cronbach’s alpha.

Results

A total of 88 cases and 33 controls, aged between 6 and 16 years old, completed the questionnaire. The Pedi-IKDC showed adequate test-retest reliability (interclass correlation coefficient (ICC =0.89), standard error of measurement (SEM= 5.45), smallest detectable change (SDC=15.11), and appropriate internal consistency (Cronbach alpha= 0.7). The Pedi-IKDC was also able to distinguish between patients and controls (P<0.0001).

Conclusion

The Moroccan-Arabic version of the Pedi-IKDC showed acceptable psychometric properties and can be used in children with knee disorders.

## Introduction

The knee is a common source of orthopedic complaints in children and adolescents. These problems include fractures, meniscal injuries, and osteoarticular infections.

Knee disorders often result in motor deficits, pain, and lack of function and lead to a decrease in sports activity [1], which can have a negative impact on health-related quality of life (HRQOL) [2-4], hence the need for valid scales for the evaluation of knee function in children and adolescents.

The evaluation of knee pathologies uses various patient-reported outcome measures (PROMs). These are subjective, patient-administered questionnaires that have become widely used in sports medicine research, such as the International Knee Documentation Committee (IKDC) and the Knee Injury and Osteoarthritis Outcome Score (KOOS). The same tools used in cases of adults have been used in children, but recent studies [5,6] have shown them to be inappropriate for children. These scales include technical words that are difficult to understand, and the questionnaires are very long for a child's attention capacity, which results in inaccurate data. In this effect, various scales have been developed to evaluate the impact of knee disorders in children, for example, the KOOS Child, the Pediatric Functional Activity Brief Scale (Pedi-FABS), and the Pediatric IKDC (Pedi-IKDC).

Pedi-IKDC is an adapted version of the IKDC questionnaire for pediatric patients. It was developed to measure pain, symptoms, and functioning in daily and sports activities in children with knee disorders. It has been translated and validated in several languages and has shown the best psychometric properties compared to other instruments [7]. In Morocco, there is no valid score for the assessment of knee function in children. Thus, the interest of the present study, which had the following objective, was to validate the Pedi-IKDC score in the Moroccan pediatric population.

## Materials and methods

Pedi-IKDC questionnaire

The IKDC Subjective Knee Forms were modified to develop a new version for use with children and adolescents (Pedi-IKDC). It was designed to measure symptoms and functional limitations in sports activities caused by various knee impairments. It consists of four VAS-based questions and 17 questions with a Likert scale. The Likert scales ranged from 1 to 5 for fifteen questions and from 1 to 2 for two questions. After transformation, the score ranges from 0 to 100. A score of 100 is interpreted as no limitation in sporting activities or daily living and the complete absence of symptoms [8].

Translation procedure

The Pedi-IKDC was translated from its original English version to the Arabic dialect in accordance with the guidelines for cross-cultural adaptation of self-report measures reported by Beaton et al. [9]. The original Pedi-IKDC was translated by two separate translators into Moroccan Arabic. At a meeting, including the two translators and the epidemiology research group, the two forward translations were combined to create the initial version of the Arabic Pedi-IKDC. Two other translators with good English language skills who had never seen the Pedi-IKDC questionnaire previously were brought in to complete the subsequent back translation into English. The original version and this version were compared. The Pedi-final IKDC draft was approved in Moroccan Arabic. The pre-final version of the Pedi-IKDC was then pilot-tested in 10 kids with various knee problems to verify that it was comprehensible and acceptable for the target group after this thorough cultural modification. Participants were invited to complete the questionnaire and provide feedback on any questions that were unclear in the translated form. The final Moroccan version of the Pedi-IKDC questionnaire was obtained after these issues were considered, noted, and modified as necessary.

Clinical study

The study was conducted at the Pediatric Orthopedic Surgery Department in 2021-2022. Moroccan Arabic dialect-speaking children, aged between 6 and 16 years old and affected by knee pathologies, were included in the study. Control participants of the same age with no lower extremity pathology (past or current) were also recruited. Parents were given full information about the study, and children whose parents gave consent were included in it. Patients or controls who did not speak the Arabic dialect and those with cognitive or mental difficulties that might affect their response to the Pedi-IKDC were excluded. The Streiner curve [10] was used to determine the sample size for an ICC of 0.70 and a precision of ± 0.10. Based on this curve, a minimum sample size of 100 participants is recommended.

The participants were asked to answer a questionnaire comprising sociodemographic data and the translated Arabic version of the Pedi-IKDC questionnaire. Parents were allowed to help children complete the form, if necessary. The Moroccan Arabic version of the Pedi-IKDC was administered a second time to patients after 15 days.

Data analysis and psychometric tests

The Pedi-IKDC score was calculated using the scoring guidelines [8]. Responses to each question are scored using the ordinal method, assigning a score of 0 to responses that represent the lowest level of function or the highest level of symptoms. The total Pedi-IKDC is scored by summing the scores for the individual items. The raw score varies between 0 and 92. After transformation, the score ranges from 0 to 100 [8]. The normality of the Pedi-IKDC score was tested by the Kolmogorov test. Descriptive statistics (frequencies, means, and standard deviations) were used for the description of scores, sociodemographics, and clinical variables. The internal consistency of the questionnaire was calculated using the Cronbach alpha. Acceptable values of alpha range from 0.70 to 0.95 [11]. The test-retest reliability was assessed by the interclass correlation coefficient (ICC) and using parameters of measurement error: the Standard Error of the Mean (SEM) and the Smallest Detectable Change (SDC). Values of ICC greater than 0.75 are indicative of good reliability [12]. The error parameters were calculated as follows: SEM= standard deviation (SD) difference/√number measurements), and SDC = SEM × 1.96 × √2) [13]. The discriminative ability of the Pedi-IKDC questionnaire in the case group was explored by comparing the score in relation to the following variables: gender (male/female), age groups (pediatric/adolescent), disease types, and between cases and controls, to test specific hypotheses formulated by a panel of experts. Differences were assessed using the Mann-Whitney and Kruskall-Wallis tests. All statistical analyses were performed by IBM Corp. Released 2019. IBM SPSS Statistics for Windows, Version 26.0. Armonk, NY: IBM Corp.

Ethics statement

The study was approved by the Ethics Committee of the Hassan II University Hospital (N 07/19). The parents of participants provided informed consent before enrollment.

## Results

Socio-demographic data

A total of 130 children (88 cases and 44 controls) were included in the study. The mean age was 9.82±3.15, 63.1% were male, and 22.4% were not in school. There was no significant difference between cases and controls in terms of socio-demographic characteristics. Demographic data of the patients and controls are shown in Table 1. 

**Table 1:** Description of population

	Total (n=130)	Cases (n=88)	Controls (n=44)	p value
Age	9.82±3.15	9.6±3.1	10.2±3.2	0.26
Age group				
Pediatric (6-12 y)	102(78.5)	71(69.6)	31(30.4)	0.37
Adolescent (13-16 y)	28(21.5)	17(60.7)	11(39.3)	
Gender				
Boys	82(63.1)	24(29.3)	58(70.7)	0.33
Girls	48(36.9)	18(37.5)	30(62.5)	
Education level				
Not in school	29(22.4)	6(20.7)	23(79.3)	
Primary	68(54.8)	24(35.3)	44(64.7)	0.31
Secondary	27(21.8)	10(37.0)	17(63.0)	
Type of disease				
Trauma	-	30(35.7)	-	
Infection	-	24(28.6)	-	
Malformation	-	26(31.0)	-	
Tumor	-	4(4.8)	-	

Description of score

In total, patients completed 130 Q1 surveys and 86 Q2 surveys. The total scores obtained from the surveys were: 58.54±15.75 for Q1 and 60.22± 16.03 for Q2 Pedi-IKDC.

Acceptability

The average time to complete the Pedi-IKDC was 3.5 min. The missing data rate for items ranges between 0 and 2.3%.

Internal consistency

The analysis of internal consistency demonstrated a Cronbach alpha of 0.7, indicating a high correlation between the items in the questionnaire.

Reproducibility

The tool demonstrated good reliability; the ICC was 0.89. The SEM and SDC were 5.45 and 15.11, respectively.

Discriminant validity

The discriminant capacity is explored in Table 2. The Pedi-IKDC was able to distinguish between patients and controls. The score was higher in controls compared to cases (p < 0.0001). Our sample did not show differences in scores between boys and girls, age groups, or the different types of disease.

**Table 2:** Discriminant capacity of Pedi-IKDC score

Hypothesis tested		Pedi-IKDC score (mean±SD)	p-value
Age groups (pediatric or adolescent) will not have a significant impact on the total score	Age group		0.65
	Pediatric (6-12 y)	54.47±15.92	
	Adolescent (13-16 y)	53.90±16.19	
Girls will have a worse score than boys	Gender		0.44
	Boys	55.47±15.65	
	Girls	52.21±16.37	
Controls will have a best score than cases	Cases/controls		<0.0001
	Cases	54.36±15.88	
	Controls	67.31±11.34	
Type of disease will not have a significant impact on the total score	Type of disease		0.16
	Trauma	56.84±16.98	
	Infection	47.50±18.38	
	Malformation	54.72±13.07	
	Tumor	61.68±5.35	

## Discussion

This study aimed to assess the validity and reliability of the Moroccan Arabic version of the Pedi-IKDC questionnaire. The results showed that the questionnaire is a feasible and adequate instrument to measure knee function and symptoms in a pediatric Moroccan population. This is illustrated by the low number of missing data, the short time required for conducting the interviews, the excellent test-retest reliability, the good internal consistency, and the discriminative ability to distinguish between cases and controls. 

In the present study, the scores in the cases were similar to those found in the Danish [14] and English studies [15] and lower than those obtained in the study of Italian children [16]. In the control group, the score was lower than the score found in the normal population [17].

This difference can be explained by the fact that the controls in our study were hospitalized patients, even if they did not have knee pathology.

The study demonstrated that the test/retest correlations of the Arabic version of the questionnaire were excellent (ICC=0.89). Similarly, studies [14,16,18,19] reported excellent reliability of the Pedi-IKDC with an ICC range of 0.9 to 0.96. 

The Moroccan version of Pedi-IKDC demonstrated a good correlation between the different items in the form (Cronbach alpha= 0.7). Other validation studies [14,16,18,20,21] also showed good internal validity.

Our results showed that the Moroccan version of the Pedi-IKDC had a good capacity to discriminate between patients and controls. In the study of Nasreddine et al. [17], the Pedi-IKDC was able to distinguish patients with and without a history of knee surgery.

The comparison of scores according to participant characteristics (sex and age) did not show a significant difference. In line with this result, Nasreddine et al. [17] did not find a significant difference in scores between the two sexes.

This study is the first to our knowledge to validate the Pedi-IKDC in Morocco. The Pedi-IKDC was cross-cultural and translated according to international guidelines. In addition, the study included children and adolescents with different knee problems. However, it has some limitations: First, the study did not measure criterion and construct validity. Second, the responsiveness to change, which is defined as ‘’the ability to detect clinical changes between the patient’s pre-intervention and post-intervention states’’ [16], is not evaluated.

## Conclusions

In conclusion, the Arabic-Moroccan version of the Pedi-IKDC has demonstrated acceptable psychometric properties and can be used in the pediatric population. The use of the Moroccan Arabic version of the questionnaire in the evaluation of knee function in our population will allow a reliable comparison with international results, and it is also a criterion of effectiveness in clinical trials based on quality of life as a criterion of judgment as well as in cost-utility studies.

## References

[1] Francavilla ML, Restrepo R, Zamora KW, Sarode V, Swirsky SM, Mintz D (2014). Meniscal pathology in children: differences and similarities with the adult meniscus. Pediatr Radiol.

[2] Coburn SL, Barton CJ, Filbay SR, Hart HF, Rathleff MS, Crossley KM (2018). Quality of life in individuals with patellofemoral pain: A systematic review including meta-analysis. Phys Ther Sport.

[3] Marson BA, Craxford S, Deshmukh SR, Grindlay DJ, Manning JC, Ollivere BJ (2020). Quality of patient-reported outcomes used for quality of life, physical function, and functional capacity in trials of childhood fractures. Bone Joint J.

[4] Valovich McLeod TC, Bay RC, Parsons JT, Sauers EL, Snyder AR (2009). Recent injury and health-related quality of life in adolescent athletes. J Athl Train.

[5] Iversen MD, Lee B, Connell P, Andersen J, Anderson AF, Kocher MS (2010). Validity and comprehensibility of the International Knee Documentation Committee Subjective Knee Evaluation form in Children. Scand J Med Sci Sports.

[6] Örtqvist M, Roos EM, Broström EW, Janarv PM, Iversen MD (2012). Development of the Knee Injury and Osteoarthritis Outcome Score for children (KOOS-Child): comprehensibility and content validity. Acta Orthop.

[7] Dietvorst M, Reijman M, van Groningen B, van der Steen MC, Janssen RP (2019). PROMs in paediatric knee ligament injury: use the Pedi-IKDC and avoid using adult PROMs. Knee Surg Sports Traumatol Arthrosc.

[8] (20229202261). Scoring Instructions for the Pedi-IKDC Subjective Knee Evaluation Form. Internet.

[9] Beaton DE, Bombardier C, Guillemin F, Ferraz MB (2000). Guidelines for the process of cross-cultural adaptation of self-report measures. Spine.

[10] Streiner D, Norman G (2015). Health Measurement Scales: A Practical Guide to Their Development and Use.

[11] Tavakol M, Dennick R (2011). Making sense of Cronbach's alpha. Int J Med Educ.

[12] Koo TK, Li MY (2016). A guideline of selecting and reporting intraclass correlation coefficients for reliability research. J Chiropr Med.

[13] Hopkins WG (2000). Measures of reliability in sports medicine and science. Sports Med.

[14] Jacobsen JS, Knudsen P, Fynbo C, Rolving N, Warming S (2016). Reproducibility and responsiveness of a Danish Pedi-IKDC subjective knee form for children with knee disorders. Scand J Med Sci Sports.

[15] Oak SR, O'Rourke C, Strnad G (2015). Statistical comparison of the pediatric versus adult IKDC subjective knee evaluation form in adolescents. Am J Sports Med.

[16] Macchiarola L, Grassi A, Di Paolo S (2020). The Italian cross-cultural adaptations of the paediatric International Knee Documentation Committee Score and the Hospital for Special Surgery Paediatric Functional Activity Brief Scale are reliable instruments in paediatric population. Knee Surg Sports Traumatol Arthrosc.

[17] Nasreddine AY, Connell PL, Kalish LA, Nelson S, Iversen MD, Anderson AF, Kocher MS (2017). The pediatric International Knee Documentation Committee (Pedi-IKDC) subjective knee evaluation form: normative data. Am J Sports Med.

[18] Herrera Rodríguez JS, Ponce de León MC, Castañeda JF, Yela H, Díaz A (2022). Transcultural validation and adaptation of the Pedi-IKDC scale for the functional assessment of children that undergo knee surgery. Rev Esp Cir Ortop Traumatol.

[19] van der Velden CA, van der Steen MC, Leenders J, van Douveren FQ, Janssen RP, Reijman M (2019). Pedi-IKDC or KOOS-child: which questionnaire should be used in children with knee disorders?. BMC Musculoskelet Disord.

[20] Ivanov IA, Александрович ИЯ, Eltsin AG, Геннадьевич ЕА, Mininkov DS, Сергеевич МД (2021). Validation and cultural adaptation of the Russian version of the Pedi-IKDC questionnaire. Pediatric Traumatology, Orthopaedics and Reconstructive Surgery.

[21] Kocher MS, Smith JT, Iversen MD (2011). Reliability, validity, and responsiveness of a modified International Knee Documentation Committee Subjective Knee Form (Pedi-IKDC) in children with knee disorders. Am J Sports Med.

